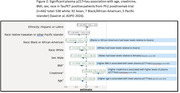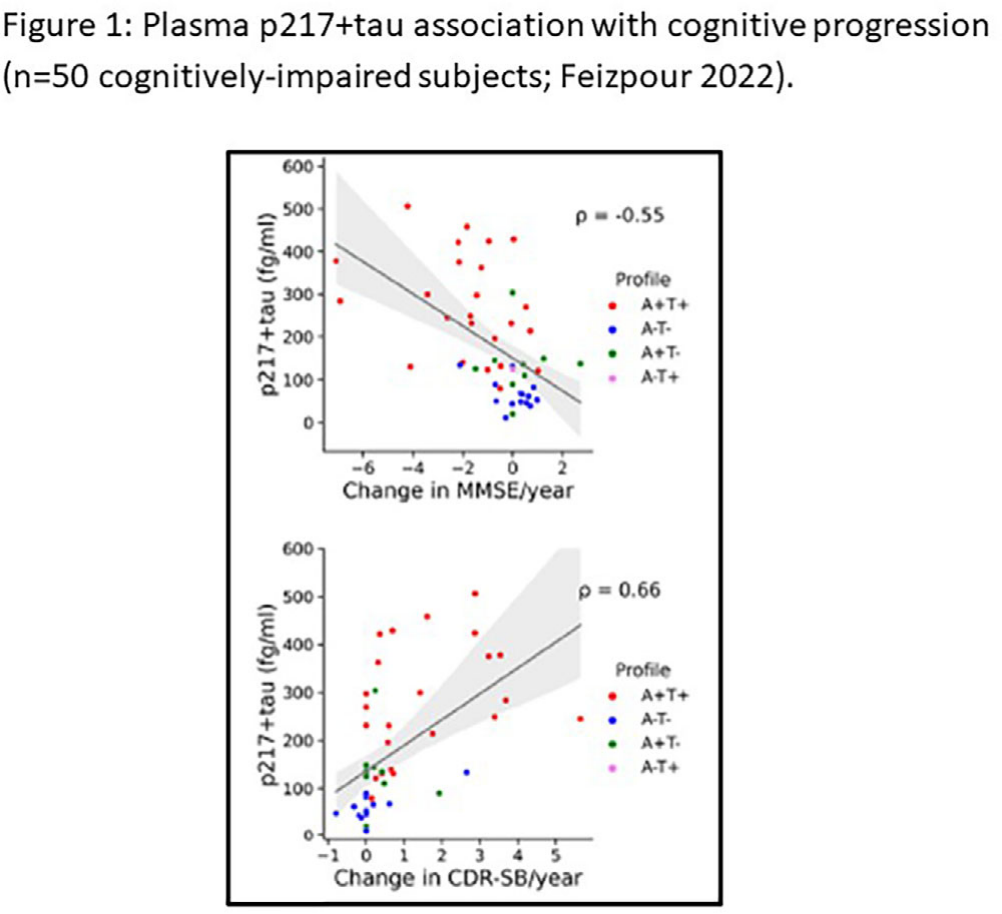# Development, Validation, and Clinical Utility of the Janssen plasma p‐tau 217+tau Simoa assay

**DOI:** 10.1002/alz.094604

**Published:** 2025-01-09

**Authors:** Gallen Triana‐Baltzer

**Affiliations:** ^1^ Johnson and Johnson Innovative Medicine, San Diego, CA USA

## Abstract

**Background:**

Over the last two decades the Alzheimer’s biomarker field has progressed dramatically, beginning with the identification of specific analytes in CSF that track with autopsy‐confirmed AD, progressing to development of specific PET tracers and CSF assays to monitor brain amyloid and tau burden up to 20 years before symptoms, and now delivery of highly accurate, sensitive, and precise assays for measuring specific Abeta and pTau species in plasma.

**Method:**

Here we present on the development, validation, and clinical utility of Janssen’s plasma p217+tau Simoa assay. The assay was initially created for CSF in 2017 and used as a pharmacodynamic marker in the Ph1 posdinemab trials, demonstrating substantial dose dependent reduction in CSF p217+tau. Translation of the assay to plasma required several key modifications, to enable quantification of the much lower levels in the periphery (∼1% of CSF levels) and avoid substantial negative interference in plasma (Triana‐Baltzer 2021).

**Result:**

Over the past four years the Janssen plasma p217+tau assay has been evaluated in >10 large clinical cohorts, where it has consistently demonstrated high accuracy in MCI/AD populations for identifying brain amyloid or tau status (by CSF or PET; AUCs 0.85‐0.95; e.g. Dore 2022) as well as cognitive decline (Fig. 1, Feizpour 2023; Saloner 2023). Cutoff concentrations and specificity/sensitivity parameters appear conserved when transitioning from symptomatic to cognitively unimpaired populations (Dore 2022), however more work is needed to confirm this. Preliminary data indicates age, creatinine, BMI, sex and/or race may impact p217 concentrations (Fig. 2, Pichet‐Binette 2023), however more work is needed.

In late 2023 the assay was validated at Quanterix as a Lab Developed Test (LDT) in CAP/CLIA environment for global diagnostic availability. This test, marketed as Lucent AD p217, was evaluated in two large cohorts (n = 352 and 521, MCI/AD) against amyloid status (PET or CSF). The assay captured 100% of samples in the linear range and employs a 2‐cutoff approach for identifying subjects with low/intermediate/high risk of amyloid positivity (ala Brum 2023), with observed 90.3% sensitivity, 91.3% specificity for identifying low and high‐risk groups

**Conclusion:**

To accelerate clinical trials the assay is currently being used as a prescreening tool in multiple Janssen‐sponsored Ph2 trials.